# PathwayVote: an R package for robust pathway enrichment analysis for DNA methylation data using a consensus-based voting framework

**DOI:** 10.1093/bioinformatics/btaf590

**Published:** 2025-10-27

**Authors:** Yinan Zheng, Feng Gao, Lifang Hou

**Affiliations:** Department of Preventive Medicine, Northwestern University Feinberg School of Medicine, Chicago, IL 60611, United States; Fielding School of Public Health, University of California, Los Angeles, Los Angeles, CA 90095, United States; Department of Preventive Medicine, Northwestern University Feinberg School of Medicine, Chicago, IL 60611, United States

## Abstract

**Motivation:**

Pathway enrichment analysis is commonly used to interpret epigenomewide association studies, yet conventional methods often rely on arbitrary thresholds and simplified CpG–gene mappings, making them sensitive to analytical choices and unable to fully leverage CpG–gene relationships Recent advances in expression quantitative trait methylation (eQTM) studies offer a rich resource to refine these mappings, but are rarely utilized in DNA methylation enrichment pipelines.

**Results:**

We developed PathwayVote, an R package that implements a voting-based consensus approach and leverages eQTM data to identify robustly enriched pathways. PathwayVote reduces dependence on arbitrary cutoffs and improves sensitivity and reproducibility of enrichment results.

**Availability and implementation:**

PathwayVote is freely available on GitHub (https://github.com/YinanZheng/PathwayVote) under the GPL-3 license and CRAN: https://CRAN.R-project.org/package=PathwayVote. The version of the code corresponding to this manuscript has been archived on Zenodo (https://doi.org/10.5281/zenodo.17209507).

## 1 Introduction

Functional interpretation of DNA methylation biomarkers identified from epigenome-wide association studies (EWAS) is essential for elucidating the molecular mechanisms underlying complex traits. Pathway enrichment analysis is an efficient bioinformatic analysis that can offer guidance for downstream experimental validation and translational applications (Reimand *et al.* 2019). However, this analysis remains challenging in DNA methylation studies due to the complexity of CpG–gene regulatory relationships and the interpretations are sensitive to user-defined parameters, limiting reproducibility and biological interpretability in epigenetic studies (Boison *et al.* 2022).

A critical foundation of pathway enrichment analysis for DNA methylation data is the proper mapping of CpG sites to their target genes. These CpG–gene relationships may reflect local transcriptional repression via promoter methylation (Moore *et al.* 2013), but also frequently involve long-range mechanisms such as chromatin looping (Popay and Dixon 2022) and enhancer–promoter interactions (Whalen *et al.* 2016). As a result, a single CpG site may regulate multiple genes, and a single gene may be influenced by multiple CpGs, forming a many-to-many relationship. Most existing approaches reported in the literature rely on assigning each CpG to its nearest gene based on genomic proximity. However, this assumption is biologically simplistic and often misrepresent CpG–gene linkage, introducing biases into the pathway enrichment analysis and misleading interpretation.

The accumulation of large-scale transcriptomic datasets in human cohorts, such as those from the NHLBI Trans-Omics for Precision Medicine (TOPMed) project (Taliun *et al.* 2021), has enabled the systematic identification of empirical many-to-many CpG-gene relationships, i.e. expression quantitative trait methylation (eQTM) studies (Keshawarz *et al.* 2023). However, existing pathway enrichment frameworks typically consider the strongest CpG–gene pairings, neglecting the broader network of weaker but potentially biologically relevant associations, thereby failing to fully leverage the complexity of eQTM data.

To address these limitations, we developed PathwayVote, a novel R package and enrichment algorithm explicitly designed for eQTM-informed methylation enrichment analyses. PathwayVote systematically evaluates pathway enrichment across multiple combinations of top-ranked CpG subsets and eQTM filtering parameters, which are determined with data-driven approaches. Inspired by ensemble learning principles, PathwayVote aggregates enrichment results through a voting-based consensus framework, prioritizing pathways consistently enriched across parameter combinations. This approach is designed to reduce dependence on arbitrary cutoffs, capture both strong and subtle regulatory signals, and improve the robustness of biological interpretation in methylation studies.

## 2 Methods

### 2.1 Overview

**Figure 1.**
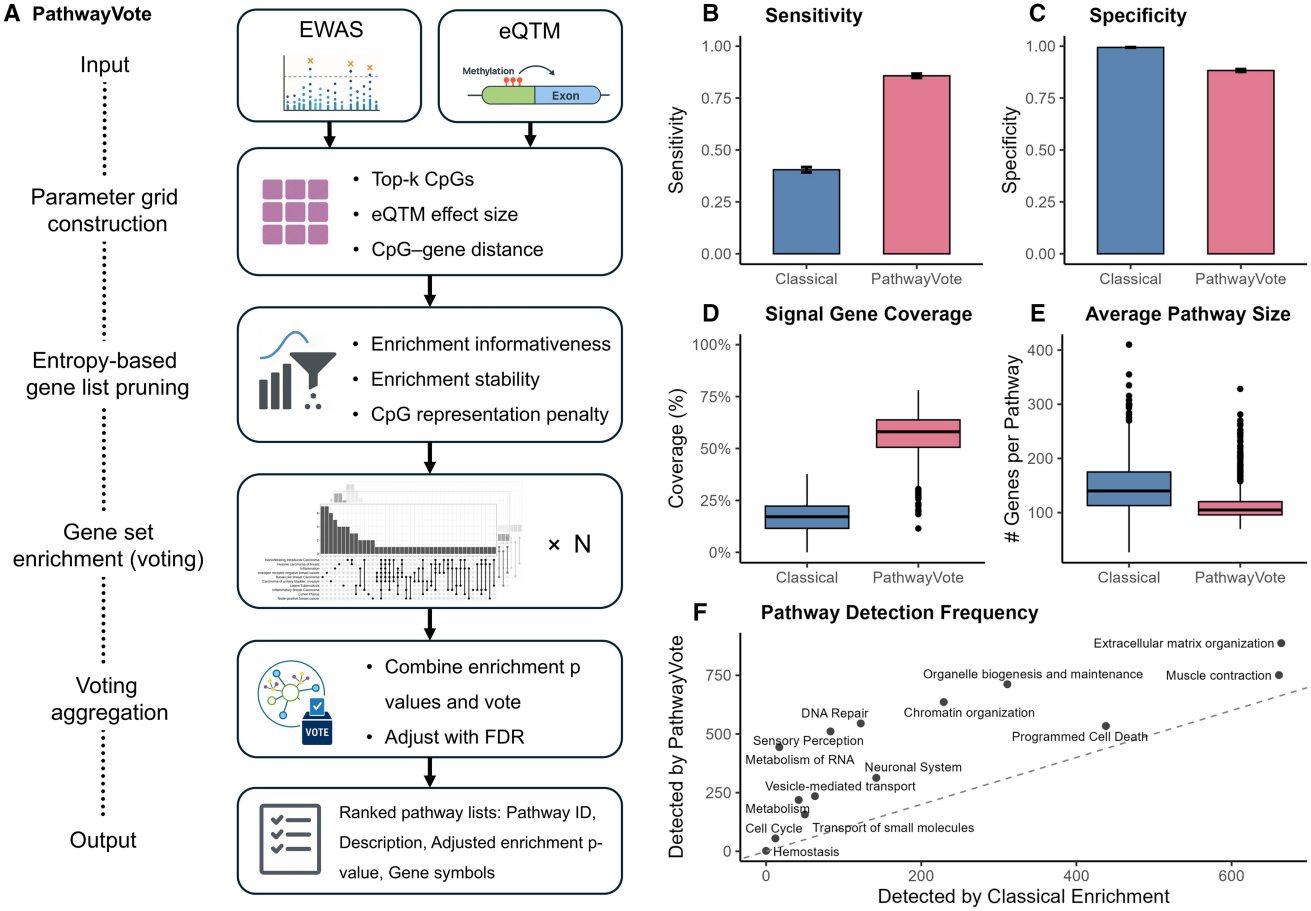
Overview and performance comparison of PathwayVote versus classical enrichment methods. (A) Schematic overview of the PathwayVote algorithm. PathwayVote constructs a parameter grid across key CpG–gene mapping thresholds, generates gene lists, prunes them using an entropy-based framework (balancing informativeness, stability, and gene-level CpG representation penalty), performs parallel enrichment analyses, and aggregates results via a voting and harmonic mean *P*-value strategy. (B) PathwayVote achieved 2.1 times higher sensitivity, identifying a larger proportion of true signal pathways. (C) Specificity was maintained at comparable and high levels (88% versus 99%), indicating controlled false positive detection. (D) Signal gene coverage, i.e. the proportion of truly regulated genes recovered in the enriched pathways, was 3.4 times higher for PathwayVote. (E) This improvement in coverage was accompanied by 24% smaller pathway sizes than those from classical enrichment, indicating more granular and interpretable results by PathwayVote. (F) Per-pathway detection frequencies (out of the 1000 simulations) across 14 ground-truth, top-level Reactome pathways show consistently higher recovery rates for PathwayVote over classical enrichment.

Figure 1A presents a schematic overview of the PathwayVote framework. Starting with EWAS results and an empirical eQTM database, PathwayVote constructs a multi-dimensional parameter grid defined by thresholds for top-ranked CpGs, eQTM effect sizes, and CpG–gene distances. For each combination, gene lists are generated and pruned using an entropy-based selection strategy that balances informativeness, stability, and gene-level CpG representation bias correction. These gene lists are then subjected to enrichment analysis, and results are aggregated using a consensus voting scheme, yielding a final ranked list of robustly enriched pathways.

### 2.2 Parameter grid construction

To ensure robust and reproducible pathway enrichment, PathwayVote constructs a grid of gene set filtering parameters. This multi-dimensional parameter space captures different biological and analytical perspectives by varying key thresholds.

- **Top-** k **CpGs**: Top-k CpGs selected based on a ranking metric (e.g. *P*-value, t-statistic, importance) derived from association studies like EWAS (stronger association is favored). When *P*-values are available, *k* values are chosen on a natural-logarithm scale between 25% and 100% of the number of CpGs with FDR <0.05. This log-spacing yields denser sampling at smaller *k* and sparser sampling near the upper bound (Fig. 1, available as supplementary data at *Bioinformatics* online) to balance between signal retention and noise exclusion.
- **eQTM effect size (** e **)**: The strength of eQTM (e.g. correlation, r-squared, t-statistic) derived from eQTM database (stronger CpG-gene linkage is favored). A grid of thresholds is generated by evenly spacing values between the 5th and 95th percentiles of the observed effect size distribution.
- **CpG-gene distance (** d **):** The genomic distance between CpG site and the transcription start site (closer CpG-gene distance is favored). Thresholds are similarly selected by evenly spacing values between the 5th and 95th percentiles of the observed distance distribution.

Note that the preference for more biologically relevant gene lists is implicitly encoded in the nested structure of the grid: stricter thresholds yield subsets of looser thresholds, which therefore tend to appear more frequently across candidate lists. While this nested design reflects biological plausibility, it can also induce frequency inflation and statistical dependency. To address these issues, we introduce additional strategies described below, including entropy-based scoring, overlap pruning, and harmonic mean *P*-value aggregation.

### 2.3 Entropy-based pruning of input gene sets

Each parameter combination in the grid (k,e, d) yields a unique gene list G. Across the grid, these lists form a collection of gene list candidates. The pruning step is to trim the candidates so that they are informative yet relatively stable across the candidates, while not dominated by genes with high CpG mapping density. The pruning step described below is done separately within each of the top *k*-CpG candidates to avoid domination by longer lists from larger *k* and preserving the hierarchical structure of CpG ranking in the final consensus.

*Information score:* To encourage diversity across candidates while avoiding length bias, we compute an entropy-style score for each candidate gene list $G_{k}$ within each *k*-specific pool. Let $N_{k}$ be the number of candidates at this k and $n_{k}(g)$ the number of candidates containing gene g. The information score is the average self-information of genes in $G_{k}$ relative to their background frequency:


$$I(G_{k})=\frac{1}{\left|G_{k}\right|}\sum_{g\in G_{k}}-\ln\left(\frac{n_{k}(g)+\alpha }{N_{k}+2\alpha }\right),\;\alpha =1$$

Here, $\left|G_{k}\right|$ denotes the number of genes in $G_{k}$. The Laplace smoothing (α=1) stabilizes the score for very rare genes. This formulation prioritizes candidates enriched for rarer genes and thus favors distinct, information-rich gene lists.

*Discordance penalty:* While $I(G_{k})$ rewards candidates that contain diverse genes, highly idiosyncratic candidates could still receive high $I(G_{k})$, even if they are poorly aligned with the majority of candidates. e counterbalance this tendency, we define a discordance penalty:


D(Gk)=1N−1∑Gk' ≠ Gk[1-J(Gk,Gk')]

where J is the Jaccard similarity $J(A,B)=\frac{\left|A\cap B\right|}{\left|A\cup B\right|}$. Higher $D(G_{k})$ indicates greater discordance with other candidate genes lists, which will be penalized.

*Gene over-representation penalty:* A gene may recur across the gene list candidates simply because many CpG sites map to that gene, rather than because it carries genuine signal. This overrepresentation may derive from assay-based platform e or from sequencing-based platform e. To down-weight this bias, we model the expected across-candidate recurrence from CpG density and penalize only the excess beyond that expectation. Let c(g) be the number of unique CpG sites mapped to gene g and set x(g)=log(1+c(g)) to stabilize the right-skewed CpG counts. Within each k-specific pool, We model the mean recurrence using a quasi-Poisson generalized linear model with a natural logarithm link: $\ln E\left[n_{k}(g)|x(g)\right]=\beta_{0}+\beta_{1}x(g)$, yielding the fitted expectation $\hat{n_{k}}(g)=\exp(\hat{\beta_{0}}+\hat{\beta_{1}}x(g))$. Quasi-Poisson accommodates over-dispersion and the heteroscedastic count structure observed (Fig. 2, available as supplementary data at *Bioinformatics* online). We then penalize only the positive “excess” recurrence. We then penalize only excess recurrence beyond this expectation using the non-negative residual $\rho_{k}(g)=\max\{0,\;n_{k}(g)-\hat{n_{k}}(g)\}$ and define the penalty as:


$$R(G_{k})=\sum_{g\in G_{k}}\ln\left(1+\rho_{k}(g)\right)$$

This construction reduces the influence of genes that recur more often than CpG density alone would predict, while not rewarding below-expected recurrence. The logarithm makes the penalty grow sublinearly so that extreme residuals do not dominate the score.

Lastly, $I(G_{k})$, $D(G_{k})$, and $R(G_{k})$ are z-scored and formed a composite score:


$$S(G_{k})=z_{I}(G_{k})-z_{D}(G_{k})-z_{R}(G_{k})$$

Candidate gene lists are then iteratively selected according to their composite scores. At each step, the list with the highest score is retained, and all remaining lists with excessive overlap (above a Jaccard similarity threshold of 0.7) are removed. The process repeats until no lists remain. This iterative pruning ensures that the final collection consists of biologically distinct, stable, and non-redundant gene sets, while mitigating artifacts from gene-level CpG overrepresentation. The choice of 0.7 as the overlap threshold is supported by simulation results, and this iterative pruning procedure ensures stable performance that is largely insensitive to the density of the parameter grid (Fig. 3, available as supplementary data at *Bioinformatics* online).

### 2.4 Pathway enrichment (“voting”)

Each pruned gene list undergoes standard enrichment analysis using Reactome, KEGG, or GO databases, leveraging the *clusterProfiler* and *ReactomePA* packages based on hypergeometric testing algorithm. While the enrichment statistic itself is count-based, the overall PathwayVote framework is ranking-informed, since it integrates nested top-k CpG subsets derived from marker rankings and aggregates them through a voting strategy. Each gene list yields a set of pathways, along with enrichment statistics. Before aggregating enrichment results, PathwayVote applies a pruning step that removes unstable or low-confidence signals: only pathways appearing in multiple enrichment runs and involving a minimum number of genes (e.g. count ≥ 2) are retained.

### 2.5 Voting aggregation

A harmonic mean *P*-value (HMP) is computed to summarize significance across the pruned enrichment results. The use of HMP to aggregate results across gene sets is statistically well-founded and particularly suitable for combining dependent tests (Wilson 2019). The algorithm returns a calibrated combined *P*-value for each pathway that remains valid under positive dependence induced by nested gene lists (R package *harmonicmeanp*). The final *P*-values across all tested pathways are then adjusted for multiple testing using the Benjamini-Hochberg method.

### 2.6 Simulation study with real eQTM database

We built an eQTM database from the publicly available NHLBI TOPMed CARDIA epigenetic (Illumina EPIC array) and transcriptomic (RNA-sequencing) studies. For each simulation run, we generated a synthetic EWAS result dataset using CpG-gene relationships from eQTM and randomly selected seven Reactome pathways from 14 top-level pathways with low redundancy (Jaccard index < 0.2) and at least 200 genes (i.e. signal pathways). Within each selected signal pathway, 25 signal genes were sampled, and up to 3 signal CpGs per gene were selected if they had strong eQTM evidence (correlation *P*-value < 1e−7). Noise CpGs (*n* = 100 000) were randomly drawn from non-signal CpGs. Each signal CpG was assigned an effect score drawn from a normal distribution (mean = 3, standard deviation = 0.5), while noise CpGs received values from a normal distribution of mean of 0 and standard deviation of 1.

As a baseline comparator, we implemented a classical enrichment method that links top-ranked CpGs to genes using the Illumina HumanMethylationEPIC annotation, which has been widely used in the literature. The genes annotated by the CpGs are used in downstream Reactome pathway enrichment using the *enrichPathway()* function from *ReactomePA* (Yu and He 2016). To account for hierarchical structure of Reactome, we considered detection of either the top-level pathway or any of its direct descendant pathways as a successful recovery of the ground-truth signal. The simulations were independently repeated 1000 times.

### 2.7 Implementation and benchmarking

PathwayVote is implemented in R with support for parallel execution. To assess runtime performance, we benchmarked typical runs under default settings on a Linux server (R 4.4.0, CentOS kernel 4.18). Using 8 workers, Reactome, GO, and KEGG enrichment completed in ∼28 s, ∼98 s, and ∼3 s, respectively, with peak RAM usage consistently <1 GB (Table 1, available as supplementary data at *Bioinformatics* online). These results demonstrate that PathwayVote is lightweight and suitable for routine use on standard workstations and high-performance computing environments.

## 3 Results

### 3.1 Higher sensitivity without significant loss of specificity

As shown in Fig. 1B and C, PathwayVote exhibited > 2 times higher sensitivity (mean 0.86, 95% CI: 0.85–0.87) than the classical enrichment approach (mean 0.40, 95% CI: 0.39–0.42), while maintaining a comparable level of specificity (PathwayVote: 0.88; Classical Enrichment: 0.99). We further examined the sensitivity on a per-pathway basis across the 14 signal pathways used in simulation (Fig. 4, available as supplementary data at *Bioinformatics* online). Some pathways were consistently easier to detect due to compact structure, whereas broader pathways posed greater challenges. Nevertheless, PathwayVote consistently achieved higher sensitivity than the classical enrichment method across all the signal pathways.

### 3.2 Enhanced true signal gene coverage and more interpretable pathway sizes

The signal pathways identified by PathwayVote exhibited greater coverage of genes (mean 56.4%) compared to those detected by classical enrichment (mean 16.7%), indicating a greater alignment with the underlying true biological signals (Fig. 1D). Notably, this gain in coverage was not achieved by inflating pathway size. On the contrary, as shown in Fig. 1E, the pathways recovered by PathwayVote were on average 24% smaller than those from classical enrichment, reflecting more granular and biologically focused gene sets rather than broad, less informative collections. This balance of higher signal gene recovery with more compact pathway sizes is enabled by the entropy-informed pruning strategy in PathwayVote, which promotes concise, stable, and non-redundant gene sets.

### 3.3 Robust detection across pathway types

Across all 14 signal pathways, PathwayVote demonstrated consistently higher detection frequencies than classical enrichment (Fig. 1F), regardless the pathway characteristics. Pathways that were missed by the classical enrichment approach were identified by PathwayVote.

### 3.4 eQTM resources

PathwayVote is platform-agnostic in design and requires only a CpG–gene mapping resource, rather than any specific array annotation. Users can construct custom eQTM resources using the *eQTM* class and *create_eQTM*() function included in the package, which automatically harmonize gene identifiers and validate input. For newer platforms where public eQTM resources may not yet exist, users can project CpGs to shared sites and leverage existing eQTM resources. As large-scale multi-omic datasets continue to grow, the availability of comprehensive and tissue-specific eQTM resources is expected to rapidly expand, further enhancing the applicability and utility of PathwayVote.

## 4 Conclusion

PathwayVote introduces a novel gene set enrichment framework for DNA methylation studies that explicitly leverages ranking information from methylation markers. It adjusts for differences in CpG density across genes and integrates evidence across pathway dimensions to produce more stable and meaningful results. By addressing the many-to-many regulatory relationships between CpGs and genes, which is often oversimplified in conventional annotation-based methods, PathwayVote provides a versatile strategy applicable to both array- and sequencing-based methylation platforms. With the continuing expansion of epigenomic and transcriptomic resources, PathwayVote is well positioned to harness these data for robust multi-omic pathway discovery. As a gene-centric enrichment tool, PathwayVote is complementary to region-centric tools [e.g. GREAT (McLean *et al.* 2010)], and future extensions could integrate eQTM-informed mappings with region-based annotations to further expand its interpretive scope.

## Supplementary Material

btaf590_Supplementary_Data

## Data Availability

The data underlying this article were accessed from Trans-Omics for Precision Medicine (TOPMed) Program (dbGaP Study Accession: phs001612).
