# Supplementary material for: PathwayVote: an R package for robust pathway enrichment analysis for DNA methylation data using a consensus-based voting framework
Source: Bioinformatics. 2025 Oct 27;41(11):btaf590. doi: 10.1093/bioinformatics/btaf590 (PMC12596613; doi:10.1093/bioinformatics/btaf590)
Supplement: btaf590_Supplementary_Data [file btaf590_supplementary_data.zip › PathwayVote - Supplementary materials_Final_Format.docx]

**SUPPLEMENTARY MATERIAL**

**for**

**PathwayVote: an R package for robust pathway enrichment analysis for DNA methylation data using a consensus-based voting framework**

Yinan Zheng^1,*^, Feng Gao^2^, Lifang Hou^1^

**Contents**

[Supplementary Table S1. 2](#_Toc211856100)

[Supplementary Figure S1. 3](#_Toc211856101)

[Supplementary Figure S2. 4](#_Toc211856102)

[Supplementary Figure S3. 5](#_Toc211856103)

[Supplementary Figure S4. 6](#_Toc211856104)

Supplementary Table S1. **Benchmarking results of PathwayVote under different pathway databases (Reactome, GO, KEGG) and parallel worker settings (2, 4, 8) on a Linux server (R 4.4.0, CentOS kernel 4.18).**

| Database | Workers | Runtime (s) | Peak RAM (MB) |
| --- | --- | --- | --- |
| Reactome | 2 | 63.6 | 302 |
| Reactome | 4 | 42.3 | 322 |
| Reactome | 8 | 28.2 | 347 |
| GO | 2 | 296.9 | 661 |
| GO | 4 | 164.3 | 622 |
| GO | 8 | 98.5 | 718 |
| KEGG | 2 | 6.3 | 273 |
| KEGG | 4 | 4.5 | 327 |
| KEGG | 8 | 2.9 | 297 |


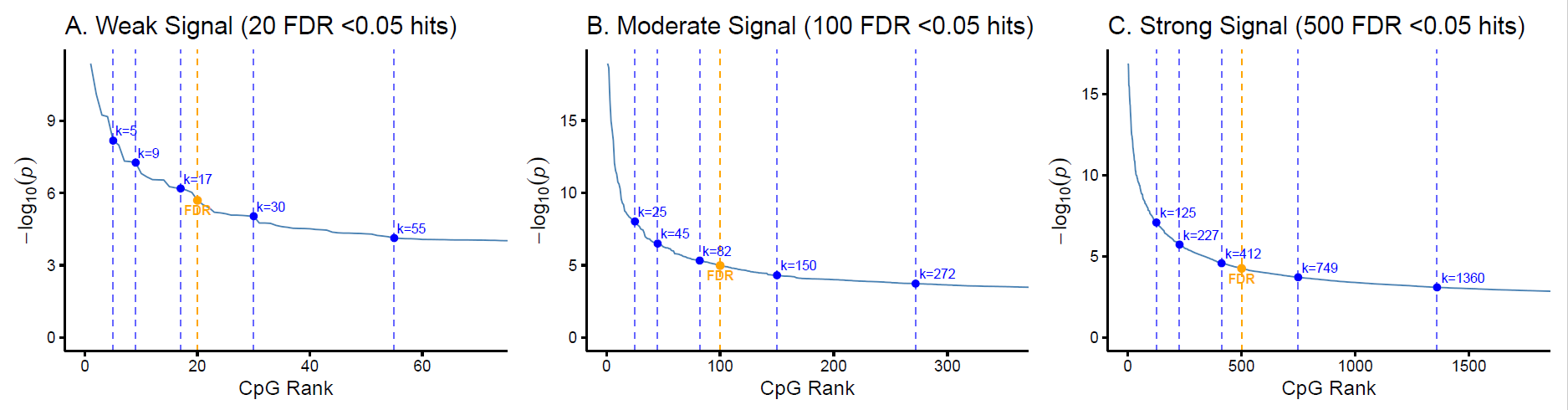


Supplementary Figure S1. **FDR-guided selection of top-ranked CpGs under different signal scenarios.**

EWAS results were simulated under three signal strength conditions: (A) weak signal (20 CpGs with FDR < 0.05), (B) moderate signal (100 FDR-significant CpGs), and (C) strong signal (500 FDR-significant CpGs). For each scenario, CpGs were ranked by increasing p-value (shown as −log₁₀(p)) and the estimated FDR was overlaid. Candidate values of k (the number of top CpGs used for gene set construction) were selected by an inflection-point detection algorithm applied to the ranked p-values, capturing the region before signal saturation.


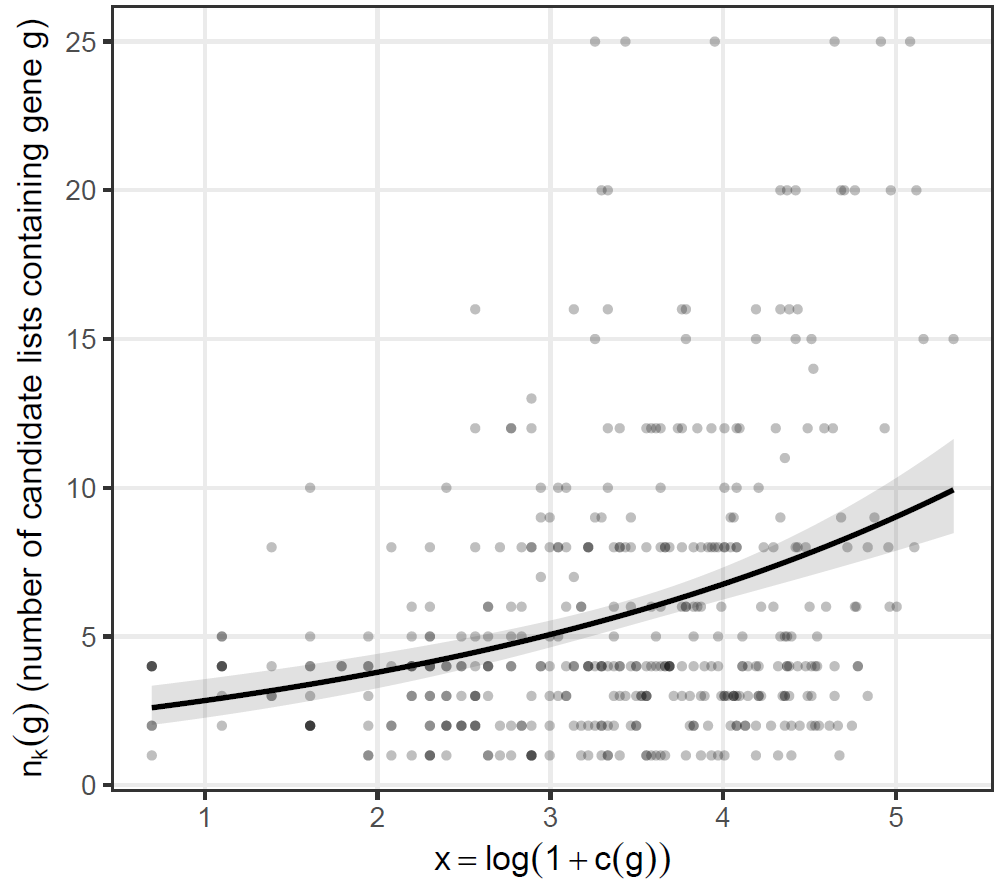


Supplementary Figure S2. **Quasi-Poisson regression of list appearance counts on CpG density per gene.**

Scatterplot shows the observed relationship between the number of candidate lists containing a gene $n_{k}\left( g \right)$ and the CpG density measure $log\left( 1+c\left( g \right) \right)$ where $c\left( g \right)$ is the number of CpGs mapped to gene $g$. The solid line represents the fitted mean from a quasi-Poisson generalized linear model with a log link, and the shaded band indicates the 95% confidence interval. This model accommodates over-dispersion and heteroscedastic count structure, illustrating that genes with higher CpG density tend to appear more frequently across candidate lists. The “excess” recurrence, i.e., points lying above the fitted expectation, are penalized in $R(G_{k})$.


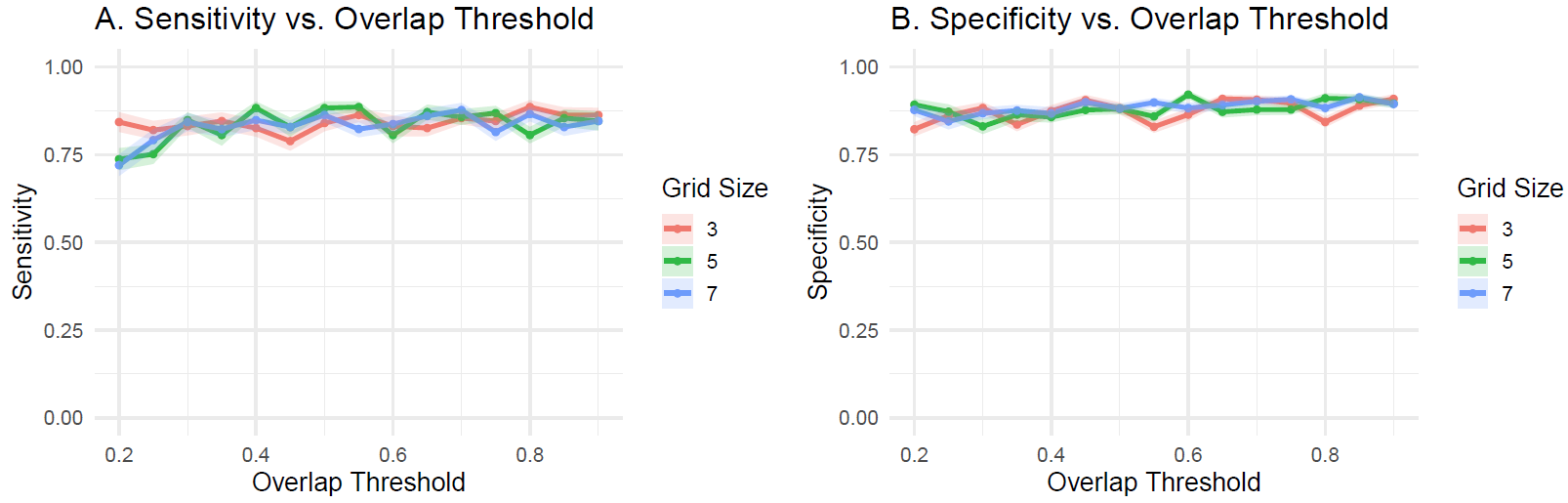


Supplementary Figure S3. **Sensitivity and specificity of PathwayVote under varying gene list overlap thresholds.**

Simulation results evaluating the performance of PathwayVote under different values of the gene list overlap threshold (x-axis), which controls the level of redundancy allowed during entropy-based pruning. (A) Sensitivity and (B) specificity are plotted separately across three grid sizes (3, 5, and 7). Performance remained robust over a broad range of overlap thresholds, with optimal balance typically achieved near 0.7. The sensitivity and specificity curves for grid sizes 3, 5, and 7 nearly overlapped, indicating that PathwayVote’s performance is not sensitive to the choice of grid size, despite the increasing number of parameter combinations (27, 125, and 343, respectively).


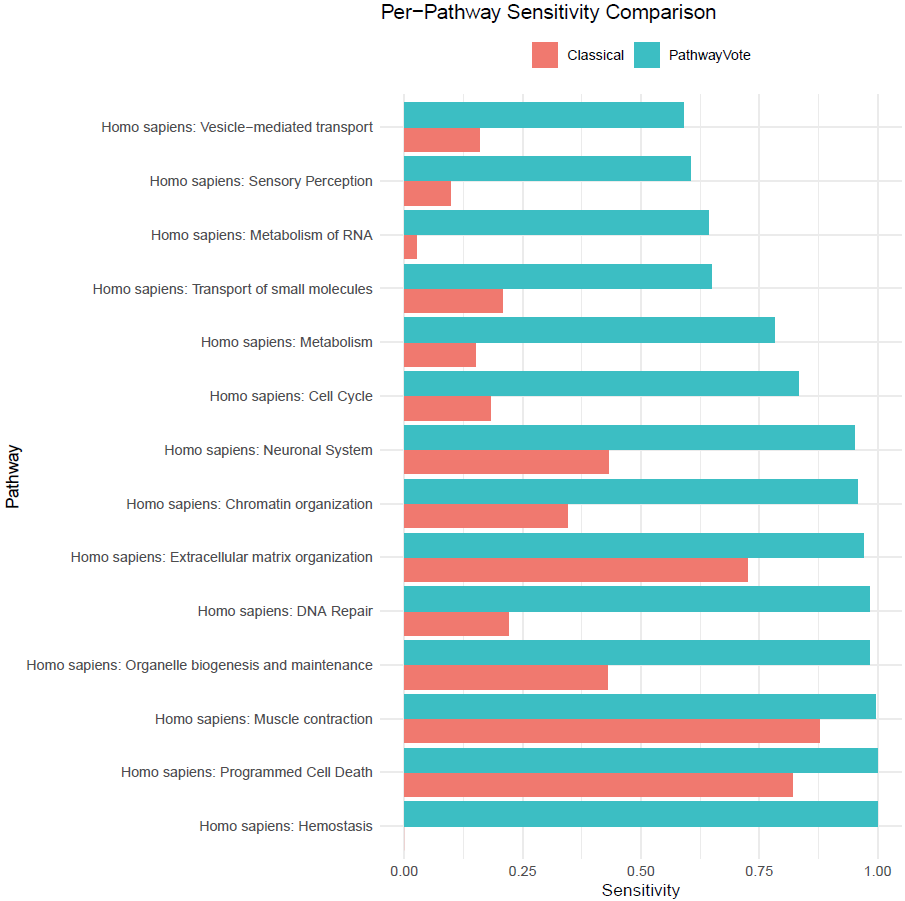


Supplementary Figure S4. **Per-pathway sensitivity comparison between PathwayVote and classical enrichment.**

For each of the 14 top-level signal pathways injected into the simulation, the proportion of replicates in which it was successfully detected (i.e., per-pathway sensitivity) is shown for both PathwayVote and the classical enrichment approach. Across nearly all pathways, PathwayVote demonstrates higher sensitivity, particularly for those with diffuse or weak signals.
